# Reversal of mitochondrial permeability transition pore and pancreas degeneration by chloroform fraction of *Ocimum gratissimum* (L.) leaf extract in type 2 diabetic rat model

**DOI:** 10.3389/fphar.2023.1231826

**Published:** 2023-11-14

**Authors:** A. J. Salemcity, John Oludele Olanlokun, A. O. Olowofolahan, F. O. Olojo, Ayodeji Mathias Adegoke, O. O. Olorunsogo

**Affiliations:** ^1^ Department of Biochemistry, University of Medical Sciences, Ondo, Nigeria; ^2^ Department of Biochemistry, Faculty of Basic Medical Sciences, College of Medicine, University of Ibadan, Ibadan, Nigeria; ^3^ Department of Pharmacology, Faculty of Health Sciences, University of the Free State, Bloemfontein, South Africa

**Keywords:** *Ocimum gratissimum* (L.), mitochondrial permeability transition pore, diabetes mellitus, glucose homeostasis, mitochondrial dysfunction

## Abstract

**Introduction:** Unmanaged Diabetes Mellitus (DM) usually results to tissue wastage because of mitochondrial dysfunction. Adverse effects of some drugs used in the management of DM necessitates the search for alternative therapy from plant origin with less or no side effects. *Ocimum gratissimum* (L.) (OG) has been folklorically used in the management of DM. However, the mechanism used by this plant is not fully understood. This study was designed to investigate the effects of chloroform fraction of OG leaf (CFOG) in the reversal of tissue wastage in DM via inhibition of mitochondrial-mediated cell death in streptozotocin (STZ)-induced diabetic male Wistar rats.

**Methods:** Air-dried OG leaves were extracted with methanol and partitioned successively between *n*-hexane, chloroform, ethylacetate and methanol to obtain their fractions while CFOG was further used because of its activity. Diabetes was induced in fifteen male Wistar rats, previously fed with high fat diet (28 days), via a single intraperitoneal administration of STZ (35 mg/kg). Diabetes was confirmed after 72 h. Another five fed rats were used as the normal control, treated with corn oil (group 1). The diabetic animals were grouped (n = 5) and treated for 28 days as follows: group 2 (diabetic control: DC) received corn oil (10 mL/kg), groups 3 and 4 were administered 400 mg/kg CFOG and 5 mg/kg glibenclamide, respectively. Body weight and Fasting Blood Glucose (FBG) were determined while Homeostasis Model Assessment of Insulin Resistance (HOMA-IR) and beta cell (HOMA-β), and pancreatic tissue regenerating potential by CFOG were assessed. Activity-guided purification and characterization of the most active principle in CFOG was done using chromatographic and NMR techniques. The animals were sacrificed after 28 days, blood samples were collected and serum was obtained. Liver mitochondria were isolated and mitochondrial permeability transition (mPT) was investigated by spectrophotometry.

**Results:** CFOG reversed diabetic-induced mPT pore opening, inhibited ATPase activity and lipid peroxidation. CFOG reduced HOMA-IR but enhanced HOMA-β and caused regeneration of pancreatic cells relative to DC. Lupanol was a major metabolite of CFOG.

**Discussion:** Normoglycemic effect of CFOG, coupled with reversal of mPT, reduced HOMA-IR and improved HOMA-β showed the probable antidiabetic mechanism and tissue regenerating potentials of OG.

## 1 Introduction

Diabetes mellitus (DM) is a metabolic derangement typified by perturbation of intermediary metabolism. Preliminary symptoms are polyuria, polydipsia, polyphagia and weight loss (Ekaiko et al., 2016). Glycosuria and ketonuria are other symptoms commonly found in children, although it could also be observed in adults. The DM has been a worrisome global health concern attributable to its attendant complications, high degree of morbidity and death (Beaglehole and Yach, 2003; Yach et al., 2004; American Diabetes Association, 2012). Long term effect of untreated DM is associated with complications which can either be microvascular or macrovascular. The former is linked to damage of the small blood vessels resulting to retinopathy, neuropathy, nephropathy, amelia-phocomelia, coma and even death. The latter is associated with disorders of large vasculatures such as atherosclerosis and cardiomyopathy (Ubaid et al., 2019). About 463 million people was reported to be living with the disease in 2019. Its prevalence in Nigeria has increased to over 6 million adults (IDF, 2019; Ugwu et al., 2020).

There are two types of DM namely, type 1 (T1DM) and 2 (T2DM). The T1DM is a disease characterised by autoimmune-dependent beta cell wreckage and lack of insulin. It is otherwise referred to as insulin dependent DM. On the other hand, T2DM is related to insulin resistance ensuing from insensitivity of the receptor to insulin in the skeletal muscle and other peripheral tissues alongside partial β-cell obliteration. It is also known as insulin non-dependent DM. Hyperglycemia, which causes several organ impairment, is a general clinical feature of DM (Feldman, et al., 2019). The T2DM accounts for about 90% of disease incidence reported (Nguyen et al., 2020).

At present, hypoglycemia drugs are employed in monitoring T2DM and these agents sometimes elicit adverse effects such as faintness, pain, dejection, sustained hypoglycemia, headache (sulphonylureas), diarrhea, vomiting, palpitation, skin rash (biguanides), oedema and cardiac arrest (thiazolidinediones) (Ganesan et al., 2020). As a result of the adverse effects associated with the use of the orthodox medicine, search for alternative with little or no side effect is necessary.

*Ocimum gratissimum* (OG) is a candidate plant which its role in the management of diabetes mellitus is being explored. *Ocimum gratissimum* belongs to Lamiaceae family, and it is widely scattered all through Africa (including Nigeria), India and some parts of south eastern Asia (Ayissi and Nyadedzor, 2003). In Nigeria, Yoruba, Hausa and Igbo tribes refer to it as “Efinrin Nla”*,* “Dai doya” and “Nchuawu” (mosquito repellant), respectively. India and Brazil call it *Vana Tulsi* and *alfavaca,* correspondingly (Effraim et al., 2000).

The plant develops to roughly 1–3 m in altitude. The dark-brown stems bear leaves which are slim and egg-shaped maturing between 5 and 13 cm in height and width of 3–9 cm. These leaves usually green in colour, possess strong aromatic fragrance similar to sweet scent of camphor. The plant grows well in lake shores, costal bush lands and in sub-montane regions (Kamboj, 2000; Alvarenga et al., 2008).

The medicinal values of the plant reside in its phytometabolites, which elicit definite physiological actions that enable it to be potent in treating malaria, dysentery, pile and lowering of blood glucose forklorically (Danziel, 1996). It has been established that phytometabolites have disease-preventing or -ameliorating capabilities and they are efficient in tackling or precluding diseases due to their antioxidant potential (Farombi, 2000).

Phytochemical investigations showed that methanol extract of OG has abundant tannins, steroids, terpenoids, flavonoids, resin, terpenoid, saponin and cardiac glycosides; and as well possesses an excellent antioxidant propensity (Afolabi et al., 2007; Okoye and Madumelu, 2013). Its leaves possess volatile essential oil which comprises majorly about 31%–66% thymol, and eugenol. It also has xanthones, terpene and lactone (Ezekwesili et al., 2004; Mohammed et al., 2007).

Experimental evidence using rats showed that its leaves extract could prevent diarrhea. It was also discovered that the methanol extract showed hepatoprotective ability in male albino rats (Salemcity et al., 2014). The OG leaf extract was also shown to have the capability to abrogate alloxan-stimulated DM in rats (Ekaiko e*t al*., 2016). This botanical drug possesses some qualities with high beneficial health relevance. These range from prevention of convulsions and seizures, reduction of high blood glucose, relaxation of intestinal muscles and anti-nociceptive use, antidotes for cough, antibronchitis and anticonjunctivitis.

Research has shown that unmanaged DM can activate the mitochondrial permeability transition (mPT) pore, leading to the release of pro-apoptotic factors such as cytochrome c into the cytosol (Daniel et al., 2018). When this pore opening is activated by some disease states and other pathological conditions, it enables the free passage of macromolecules into the mitochondria. This is preceded by factors such as calcium overload, reactive oxygen species (ROS), cytotoxic compounds, oncoproteins, DNA damage, and certain chemotherapeutic agents (such as anthracycline-doxorubicin, sunitinib, and alkylating agent-cisplatin) (Gorini et al., 2018). The mPT is accompanied by processes such as mitochondrial swelling, membrane rupture, and the release of apoptotic proteins (Wang and Youle, 2009; Olanlokun et al., 2017; Oyebode et al., 2017).

While most normoglycemic effects of *O. gratissimum* has been monitored using the leaf extract only, there is paucity of information on the purification of active principle in the most potent fraction and the probable mechanism of such metabolite to prevent tissue wastage via the modulation of the mitochondrial permeability transition pore opening. Therefore, this study aims to investigate the mechanism by which *O. gratissimum* leaf prevents hyperglycemia-induced cell death and tissue wastage in a diabetic rat model induced by streptozotocin and a high-fat diet.

## 2 Materials and methods

### 2.1 Experimental animals and treatment

Male Wistar rats each weighing between 80 and 100 g were acquired from the Veterinary Medicine Animal Holding, Department of Veterinary, University of Ibadan, Nigeria. They were conditioned for 2 weeks in Department of Biochemistry Animal Care Unit in the same institution. Water and rat chow were given *ad libitum* to the animals in a conducive environmental situations of temperature and 12-hour bright/gloom phase.

### 2.2 Induction of type 2 diabetes mellitus

Srinivasan et al. (2005) showed that T2DM could be induced in rat model by placing them on high fat diet (HFD) for 2 weeks and thereafter administered STZ. The HFD composition was presented in Table 1. Fifty rats in this study were exposed to HFD for 28 days after which a single dose STZ (35 mg/kg) dissolved in cold citrate (0.1M) buffer adjusted to pH 4.5, was administered intraperitoneally to induce diabetes mellitus (Detaille et al., 2005). After 3 days the rats underwent an overnight fast and their blood glucose was determined using glucometer (On-Call Plus^R^). Animals having blood glucose status >250 mg/dL were confirmed diabetic and ascertained suitable for further experiment. Thirty-one rats were discovered to have blood glucose level of 250 mg/dL and above. The success rate was 60%. However, ten out of the thirty-one diabetic rats died before the commencement of the treatment, with only twenty-one diabetic rats left for the experiment.

**TABLE 1:** Composition of High fat diet.

Ingredients	Diet (g/kg)
Powdered Normal Pellet Diet	365
Lard	310
Casein	250
Cholesterol	10
Vitamin-mineral mix	60
DL-methionine	3
Yeast powder	1
NaCl	1

(Srinivasan et al., 2005).

### 2.3 Grouping of animals

The normal control (fed normally with rat chow) and diabetic rats were grouped (n = 7) and treated orally once daily as follows:

Group1: Normal Control (NC) (Received corn oil)
Group 2: Diabetic control (Administered corn oil)
Group 3: Diabetic + CFOG (400 mg/kg using corn oil as vehicle)
Group 4: Diabetic + Glibenclamide (5 mg/kg using corn oil as vehicle)
Key: CFOG: Chloroform fraction of *O. gratissimum* leaf extract

### 2.4 Ethical approval

Approval for this research was obtained from Animal Care and Use Research Ethics Committee with reference number UI-ACUREC/19/0065.

### 2.5 Preparation of *Ocimum gratissimum* (L.) extract and fractions

#### 2.5.1 Chemicals

All chemicals used were analytical grade and purchased from Sigma.

#### 2.5.2 Source of plant material

*Ocimum gratissimum* (L.) was procured from “Oja-Bodija” market, Ibadan, Oyo State, Nigeria and validated in the Department of Pharmacognosy, University of Ibadan, Nigeria (Voucher specimen number: DPUI No 1504).

#### 2.5.3 Preparation of plant materials

The leaves of *O. gratissimum* (L) were air-dried at room temperature between 28–30°C for four (4) weeks and pulverized to a smooth mill with a clean grinder. The powdered leaves were kept at room temperature in a clean jar.

#### 2.5.4 Extraction and partitioning of the plant extract

Cold extraction was performed using absolute methanol in a ratio of 1:10 (w/v). The jar containing the powdered leaves and methanol was allowed to stand for 72 h. The extract was then filtered through sterile Whatman No. 1 filter paper. The green-coloured extract was concentrated using a rotary evaporator under reduced pressure. The resulting crude concentrate was further concentrated in a water bath at 37°C to obtain a solvent-free methanol extract. The final crude extract obtained, weighed 450 g from an initial dried plant sample weighing 750 g. The percentage yield of the extract was 60%. The column of the Vacuum Liquid Chromatography (VLC) was packed with silica gel for Thin Layer Chromatography (TLC) under pressure with *n*-hexane. 10 g sample of the methanol extract of *O. gratissimum* (L) was adsorbed with 10 g of the TLC gel and allowed to dry. The adsorbed sample was loaded on the VLC column and washed with *n*-hexane until exhaustion. Further to this, the column was washed with chloroform, then ethylacetate and finally with methanol successively. The fractions were concentrated using rotary evaporator and were further rendered solvent-free in a water bath. The solvent-free fractions were kept in the fridge until used.

$$\text{Percentage}\;\text{Yield}=\frac{\text{Weight}\;\text{of}\;\text{crude}\;\text{extract}}{\text{Weight}\;\text{of}\;\text{pulverised}\;\text{sample}}X\;100$$

### 2.6 Sample collection

The rats were sacrificed by cervical dislocation, the blood sample was collected into anticoagulant-free tube and centrifuged at 3,500 rpm for 5 minutes to obtain the serum. The abdominal cavity was opened up and the pancreas was excised into 10% formalin for histological studies.

### 2.7 Isolation of mitochondria from the rat liver

Reduced ionic strength mitochondria were separated using method designed by Younes and Schneider (1984), and Johnson and Lardy (1967). The rats were sacrificed by cervical dislocation and the abdominal cavity was opened up and liver samples were removed into ice-cold beaker. The blood stain was rinsed from the liver with isolation buffer that contains 0.21M mannitol, 0.079M sucrose, 0.005M HEPES-KOH and 0.001M EGTA (pH 7.4, Sigma). The samples were weighed and chopped into small pieces using a pair of scissors. This was then homogenized using Teflon and homogenizer (DELFLEX^R^) in a 10% suspension in isolation buffer. The entire process was carried out at 4°C to ensure viability of the mitochondrial membrane. The homogenate was subjected to five differential centrifugation steps in a cold centrifuge MSE. The first two-5 minutes each was used to separate the nuclear debris as the pellet at 2,300 rpm. The supernatant was then discarded, and the pellet containing the mitochondria was resuspended in a wash buffer (0.21M mannitol, 0.079M sucrose, 0.005M HEPES-KOH, pH 7.4, and 0.5% BSA, Sigma). The re-suspended mitochondria were centrifuged twice for 10 min at 12,000 rpm to wash away any artifacts. The pellet was re-suspended in suspension buffer (0.21M mannitol, 0.079M sucrose, 0.005M HEPES-KOH, pH 7.4, Sigma) and kept on ice for immediate use.

### 2.8 Determination of mitochondrial protein

Mitochondrial protein concentration was determined according to the method of Lowry et al (1951) using Bovine Serum Albumin as standard. Mitochondria (10 µL) were suspended in 990 µL of distil water in test tubes in triplicate. Then, 3 mL mixture of 100:1:1 of 2 g Na_2_CO_3_, 0.1M NaOH and 1% CuSO_4_.5H_2_O respectively, was added to the protein suspension, thoroughly mixed and left standing for 10 min. Thereafter, 300 µL of 2N Folin-Ciocalteau diluted in four-fold was added into the mixture, followed by energetic shaking and incubation for 30 min. At the expiration of the time, absorbance was determined spectrophotometrically at 750 nm.

### 2.9 The procedure and method for mPT determination

The mitochondria were first investigated to determine their suitability for this experiment. Isolated mitochondria protein (0.4 mg/mL) from normal control group were pre-incubated with 8 µM rotenone for 3.5 min. Subsequently, 5 mM succinate was added to energize the reaction and change in absorbance was read using UV-752 spectrophotometer at 540 nm for 12 min at 30 s interval. Similarly, assessment of the inductive effect of calcium was carried out as follows: mitochondria of the same protein concentration (0.4 mg/mL) were pre-incubated with 8 µM rotenone for 3 min, followed by the addition of exogenous 3 µM calcium. Thirty seconds later, 5 µM succinate was added and the absorbance was measured. The reversal of the calcium-induced opening was assessed by pre-incubating the same mitochondria protein with 8 µM rotenone and 4 mM spermine. Exogenous calcium was then added immediately after 3 min pre-incubation; succinate was added after 30 s and absorbance was read. Corresponding mitochondria protein from the treatment groups were investigated for permeability transition under similar condition without addition of exogenous calcium.

### 2.10 Assessment of mitochondrial ATPase activity

Isotonic solution (0.25M sucrose) was used to isolate mitochondria of viable integrity from rat liver in this experiment. The isolation followed the same process with that of mPT (Section 2.6) except for the buffer employed.

Mitochondrial ATPase activity was determined as described by Lardy and Wellman (1953) with minor modification. Each test tube (in triplicate) contained 25 mM sucrose, 65 mM Tris-HCl (pH 7.4) and 0.5 mM KCl in 1 mL final reaction volume. The ATP (1 mM) was added to the set of tubes labelled ATP only, uncoupler, zero time, test groups and control with the exception of the tube labelled mitochondria only. These were incubated at 27 °C in a shaking water bath. Mitochondria (0.4 mg/mL protein) from the test groups were dispensed into labelled tubes other than mitochondria only, uncoupler and zero time tubes which contained mitochondria isolated from the normal control group. While uncoupler (25 μM, 2, 4-dinitrophenol) was added to the uncoupler tubes instantly after mitochondria were added, 1 mL of 10% sodium dodecylsulphate (SDS) was immediately added to the zero-time test tubes. The suspensions were incubated for 30 min and the reaction was terminated by adding 1 mL SDS except in the zero time test tube which had been stopped before. One ml suspension was withdrawn from each test tube and diluted with 4 mL distilled water. Thereafter, 1 mL of 1.25% ammonium molybdate (prepared in 6.5% H_2_SO_4_) and 1 mL of 9% ascorbic acid newly prepared were added successively and the absorbance was read at λ_660 nm_ in a UV-752 spectrophotometer. Ammonium molybdate (1 mM) was also treated as the sample and used as the standard from which the absorbance of the unknown could be extrapolated. Inorganic phosphate released was quantified using a phosphate standard curve.

### 2.11 Determination of DNA fragmentation

Liver samples (0.25 g) from each rat were weighed and homogenized with 5 mL TET (5 mM Tris-hydroxymethyl-aminomethane, 20 mM EDTA and 2 mL of Triton X-100, adjusted to pH 8.0, Sigma) buffer and spinned using ultracentrifuge at 27,000 rpm for one-third hour. Supernatant was removed and the pellet was reconstituted to concentration of 1:1 (v/v) using TE (5 mM Tris-HCl and 20 mM EDTA pH 8.0) buffer. Exactly 0.5 mL of the supernatant and reconstituted pellet were withdrawn and 1.5 mL of 9 mM diphenyl amine solution (prepared in amber bottle because of photosensitivity) was added to each mixture. The mixture in each test tube was allowed to incubate at physiologic temperature for 16 h to 1 day for colour development to be observed and absorbance taken at 620 nm via UV-752 spectrophotometer.

$$\text{Percentage}\;\text{DNA fragmentation}=\frac{A}{A+B}X\;100$$

Where A is the absorbance of supernatant; B is the absorbance of the pellet.

### 2.12 Estimation of lipid peroxidation

A modified TBARS (thiobarbituric acid reactive substances) method was used for determining the level of peroxidation in mitochondrial membrane lipids (Ruberto et al., 2000). For this assay, exactly 2 mL of a 10% mitochondrial suspension was dispensed into a test tube and diluted with distilled water to a final volume of 4 mL. The mixture was allowed to stand at room temperature for 30 min. Subsequently, 6 mL of 20% acetic acid and 6,000 µL of 0.75% TBA in 1.1% SDS were added to the tube. The resulting solution was then subjected to steam heating for 60 min. After cooling, 5 mL of butanol was added to the solution, leading to the formation of organic and aqueous layers. The malondialdehyde (MDA), a product of lipid peroxidation, was selectively extracted into the butanol phase. The mixture was centrifuged at 3,000 rpm, resulting in a clear demarcation between the butanol and aqueous phases.

The butanol phase, containing the extracted MDA, was measured spectrophotometrically at a wavelength of 532 nm to quantify the level of peroxidation in the mitochondrial membrane.

### 2.13 Antioxidant assays

#### 2.13.1 Determination of catalase activity

It was examined as described by Claiborne (1985) protocol. This procedure is dependent on the reducing absorbance seen at wavelength 240 nm upon the action of the enzyme on H_2_O_2_. The extinction coefficient of 0.0436/mM/cm was used (Noble and Gibson, 1970). The dilution was carried out on the samples in 1:50. The reaction mixtures 2 mL H_2_O_2_ solution (19 mM) and 2.5 mL phosphate buffer (0.05 M pH 7.4). One and a half ml of the assay mixture was added into 3 mL of dichromate acetic acid reagent at 60 s periodically and then read using UV 752 spectrophotometer.

$$\text{Catalase activity}=\frac{\Delta A_{240}/\min\times \text{reaction}\;\text{volume}\times \text{dilution}\;\text{factor}}{0.0436\times \text{sample}\;\text{volume}\times \text{mg}\;\text{protein}/\text{ml}}$$

The unit is µmole H_2_O_2_/min/mg protein.

#### 2.13.2 Assessment of reduced glutathione (GSH) level

Concentration of GSH was investigated via the protocol of Beutler et al. (1963). Precipitating solution (4% sulphosalicylic acid prepared with solution containing 4 g of sodium chloride in final volume of 100 mL) and sample were in a mixture of 0.2 mL each, thoroughly mixed and centrifuged at 4,000 rpm. Subsequently, 0.25 mL of the supernatant and 0.75 mL of Ellman’s reagent (1 mM) were added. The absorbance readings were taken at 412 nm via spectrophotometer (UV- 752).

#### 2.13.3 Assessment of glutathione S- transferase activity

GST activity was estimated in accordance to Habig et al. (1974) method. The reduction in absorbance was read using UV-752 spectrophotometer at wavelength 340 nm, 30 s interval for 4 min. GST activity was calculated as unit per mg protein based on a molar extinction coefficient of 9.6 × 10^3^ L/mol/cm. One unit of GST was defined as the amount of enzyme that catalyzes the conjugation of 1 nmol of GSH-CDNB per minute.

### 2.14 Isolation and purification of active metabolites using preparative thin layer chromatography

To further purify the chloroform fraction of *O. gratissimum* leaf extract, a graded solvent system was employed using vacuum liquid chromatography, resulting in the isolation of the methanol/chloroform subfraction (1:1; v/v). Preparative thin layer chromatography plates were utilized for the analysis. Various solvent systems were employed to facilitate the elution of the samples on the plate.

To identify specific phytochemicals, chromogenic agents were applied to the separated metabolites on the plate, which was subsequently visualized under UV light (both at 254 nm and 366 nm). The samples dissolved in suitable solvents, were carefully spotted on the plate using capillary tubes. After drying, the plate was gently positioned in a chromatographic jar containing the appropriate solvent system. The jar was sealed, allowing the samples to migrate with the mobile phase. The experiment was concluded when the solvent front reached a point about 1 cm before reaching the top of the plate. The plate was then removed, air-dried, and examined under the UV light. Later, the spots on the plate were separately scraped, dissolved in the appropriate solvent and spun in the centrifuge to sediment the gel. Dissolved isolated metabolites were aspirated from the centrifuge tubes and concentrated to dryness. The samples were subjected to analysis using a nuclear magnetic resonance system (Bruker Avance^III^ 400 MH_3_).

### 2.15 Assessment of serum insulin concentration

Calibrators and samples (25 µL each) were added into the right micro-wells along with 100 μL enzyme conjugate 1X deposited in each well and shaken in a shaker at normal environmental temperature for 2 h to allow for incubation to occur. It was washed repeatedly six rounds with washing buffer 1X solution and the left-over reaction content was disposed off by inversion of the microplate. Afterwards, wash solution (WS) (350 µL) was introduced and later removed using absorbent material. The removal of WS was performed in five successive intervals. Incubation at room temperature was allowed to proceed for 15 min after addition of substrate, TMB (200 µL) to the wells. The reaction was thereafter halted with “stop solution” (50 µL) and thoroughly mixed using shaker for just 5 s. Spectrometry method via microplate reader was then employed to take the optical density reading at λ450 nm not later than duration of half-an hour.

### 2.16 Homeostasis model assessment of insulin resistance and beta cell function

These were calculated using the relationship between serum insulin and blood glucose level (Matthews et al., 1985).

$$\text{HOMA}-\text{IR}=\frac{\text{Insulin}\left(µU/\text{ml}\right)x\;\text{Glucose}\left(\text{mg}/\text{dl}\right)}{405}$$

$$\text{HOMA}-\beta =\frac{360\;x\;\text{Insulin}\left(µU/\text{ml}\right)}{\text{Glucose}\;\left(\text{mg}/\text{dl}\right)\;-63}$$

### 2.17 Hemoxylin and eosin procedure for pancreatic architecture

Wax was removed using xylene for about 15 min and passed through absolute, 95% and 70% alcohol successively. This would be followed by rinsing the section in water and staining with Harris hematoxylin for 300s. Before differentiating quickly in 1% acid alcohol, it was dipped in water again. After this, it was put under running tap for 10 min and counterstained with 1% Eosin stain for 180s, then rinsed with water. It was dried in rising grades of alcohol and cleared in xylene subsequent to mounting in DPX (Avwioro, 2010).

### 2.18 Statistical analysis

Statistical analysis was carried out using Graph pad prism (version 8.0) for one-way ANOVA and Turkey’s multiple comparison test was used to compare the mean among the groups. Level of significance was set at *p* < 0.05. All the results were expressed as mean ± standard deviation (SD). Representative profiles of absorbance of mitochondria were used for the mitochondrial permeability transition pore opening assays. Each assay was repeated three times (in each case) and representative of similar kinetic assay for the mPT in each group was used.

## 3 Results

### 3.1 Effect of the chloroform fraction of *O. gratissimum* (L) leaf extract on body weight, glycemic index and mito-protective biomarkers in STZ-triggered diabetic rats

#### 3.1.1 Effect of chloroform fraction of OG on body weight on STZ-induced diabetic rats

**FIGURE 1:**
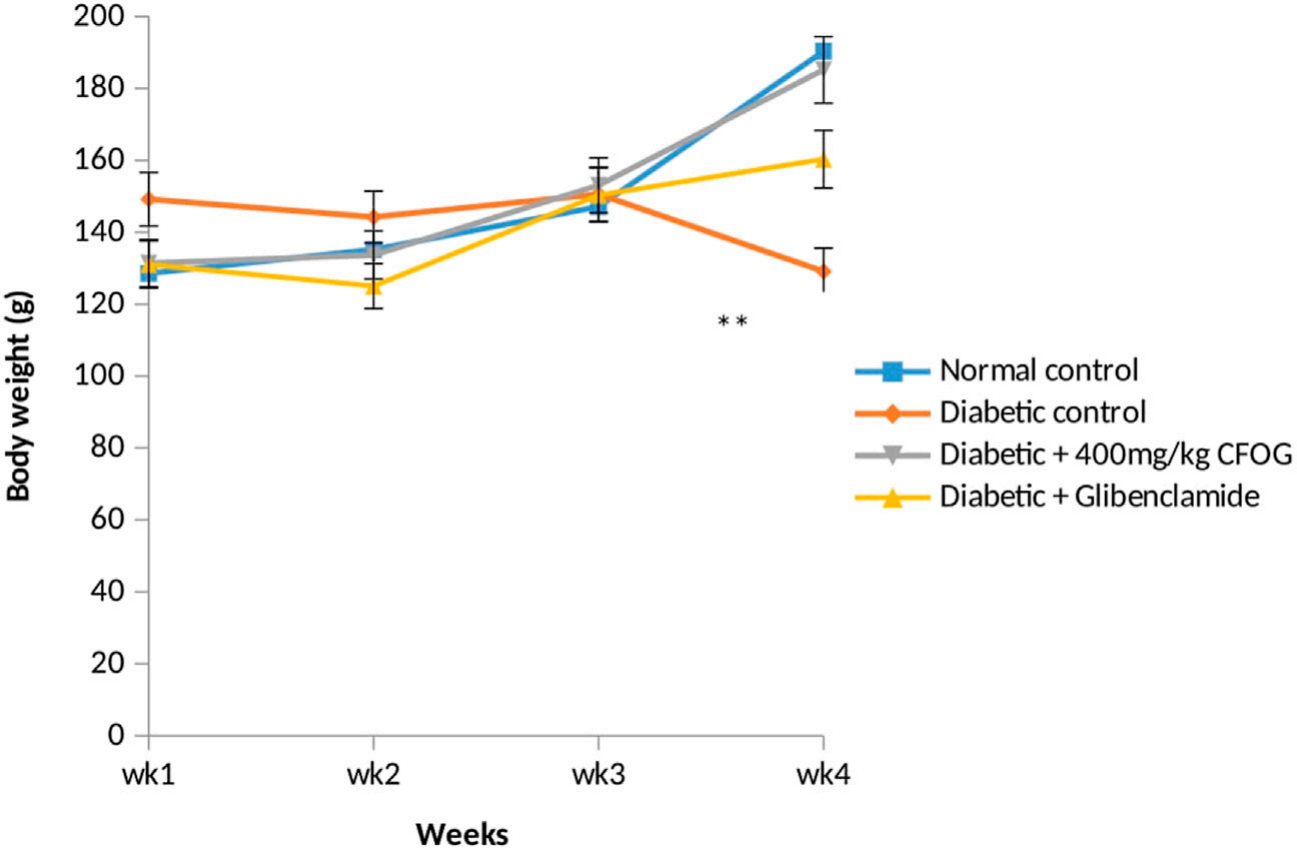
The assessment of body weight of normal and STZ-induced diabetic rats Key: CFOG: Chloroform fraction of *0. gratissim um.*

Body weight is one of the indices for tissue wastage which is common in individuals with unmanaged diabetes mellitus. Figure 1 illustrates the body mass of non-diabetic and CFOG-treated diabetic rats for a period of 28 days. It was noticed that no significant difference among the glibenclamide-, CFOG-treated animals and control. In contrast, sharp decline was observed in the diabetic untreated group when compared to control.

#### 3.1.2 Evaluation of insulin and glucose levels in CFOG-treated diabetic rats

Table 2 depicts the effect of CFOG on concentrations of glucose and insulin in STZ-stimulated diabetic rats. There was significant difference in the insulin level in untreated diabetic group relative to control. Whereas, no significant statistical change was observed in the remaining test groups compared to control. Moreover, a significant increase was observed in glucose concentration in the untreated, glibenclamide-treated and 400 mg/kg CFOG groups relative to control. However, in comparison with the diabetic control, there was obvious reduction in blood glucose level in CFOG-treated and glibenclamide groups.

**TABLE 2:** Effect of chloroform fraction of *Ocimum gratissimum* (L.) leaf extract on insulin and blood glucose concentrations in STZ- and HFD-induced diabetic rats.

Groups	Insulin concentration (μU/ml)	Blood glucose level (mg/dL)
Normal Control	1.41 ± 0.01^#^	106.74 ± 5^#^
Diabetic Control	1.74 ± 0.01*	492.3 ± 10^*^
Diabetic +400 mg/kg CFOG	1.42 ± 0.03^#^	169.92 ± 7^*#^
Diabetic +5 mg/kg Glibenclamide	1.42 ± 0.03^#^	181.08 ± 11^*#^

Key: * test groups compared to Normal control; # other groups relative to Diabetic control.

CFOG: chloroform fraction of *ocimum gratissimum* leaf extract.

### 3.2 Investigation of chloroform fraction of *O. gratissimum* (L) effect on homeostasis model assessments of insulin resistance and pancreatic beta cell function

**FIGURE 2:**
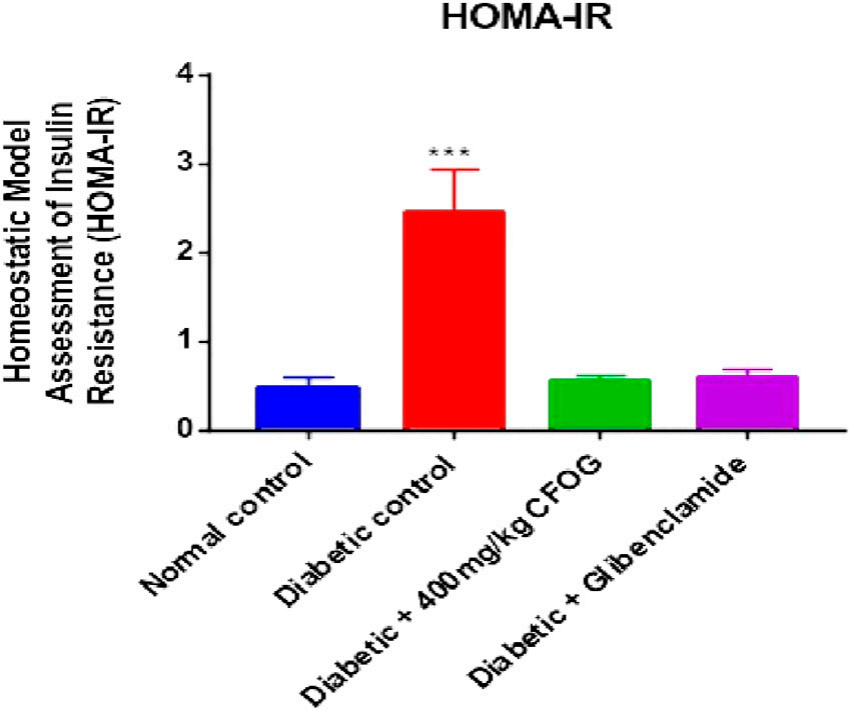
Effect of chloroform fraction of *Ocimum gratissimum* leaf on Homeostatic Model Assessment of Insulin Resistance in normal and diabetic rats.

Homeostasis model assessment is typically used in type 2 diabetes mellitus as index to measure the sensitivity of the insulin to its receptor and as well as viability and availability of beta cells that are responsible for synthesizing insulin. Figure 2 illustrates the influence of CF of *O. gratissimum* (L) leaf on insulin resistance status in STZ-induced diabetic rats. There was significant elevation of this parameter in the diabetic control relative to normal control. Conversely, no significant difference exists between the remaining treated groups compared to normal control.

**FIGURE 3:**
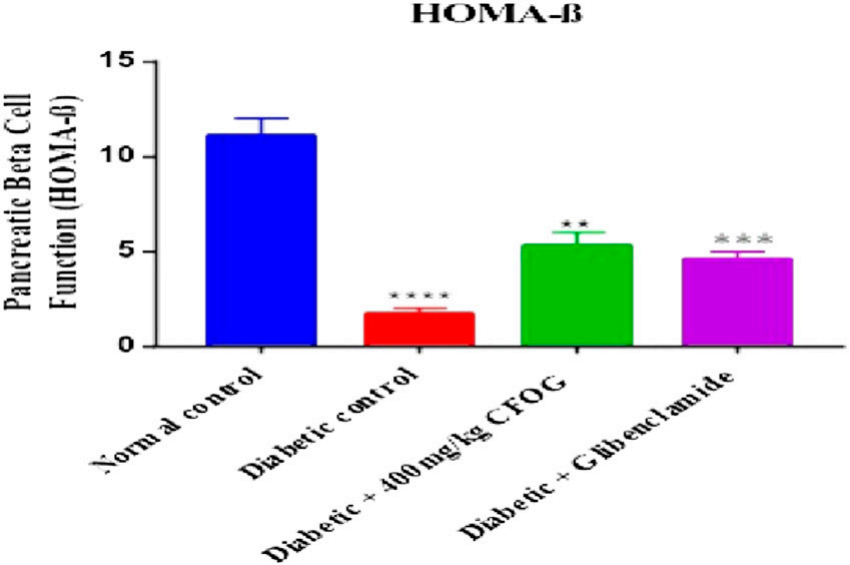
Effect of chloroform fraction on Pancreatic Beta Cell Function of STZ-induced diabetic rats.

A highly significant reduction of β-cell function was observed in the untreated group in comparison with normal control. However, there was drastic increase in beta cell status in the CFOG and glibenclamide treated groups compared to the untreated diabetic control (Figure 3).

### 3.3 Effect of chloroform fraction of OG on mPT in STZ-induced diabetic rats

**FIGURE 4:**
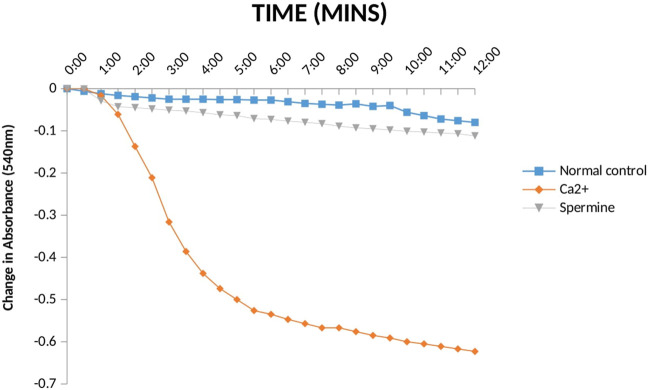
Effect Ca2+ and spermine on Mitochondrial Membrane Permeability Transition pore for normal control rats.

The status of mPT pore of the normal rats is crucial to knowing whether the pore opening in diabetic untreated rats was actually due to the administered diabetogenic agent (STZ) or not. Therefore, it is necessary to investigate the mitochondria intactness of normal control rats (non-diabetic). Figure 4 presents the evaluation of calcium induction and spermine inhibition of mPT in normal control rats. The results showed that large amplitude swelling was observed when intact mitochondria were challenged with exogenous calcium (6.3 folds), the triggering agent. However, spermine (standard inhibitor) subsequently reversed calcium-induced opening by 82%.

**FIGURE 5:**
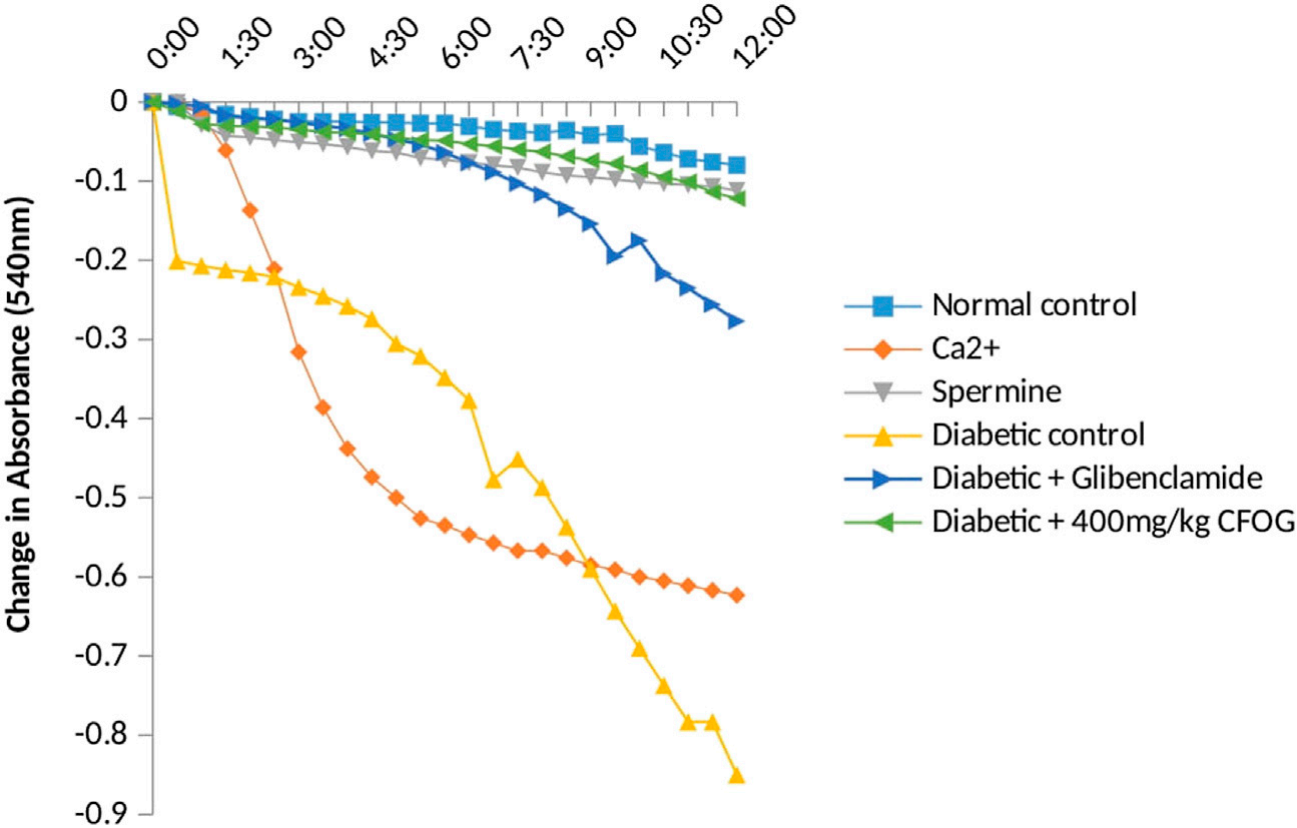
Assessment of effect of chloroform fraction of OG leaf on type 2 diabetic rat liver.

Furthermore, a significant pore induction (8.5 folds) was discovered in STZ-induced diabetic rats (Figure 5). However, CFOG and glibenclamide were able to preclude the pore opening observed in STZ-induced diabetic rats by 90% and 72% successively.

### 3.4 Assessment of the impact of CFOG on mLPO in STZ-stimulated diabetic rats

**FIGURE 6:**
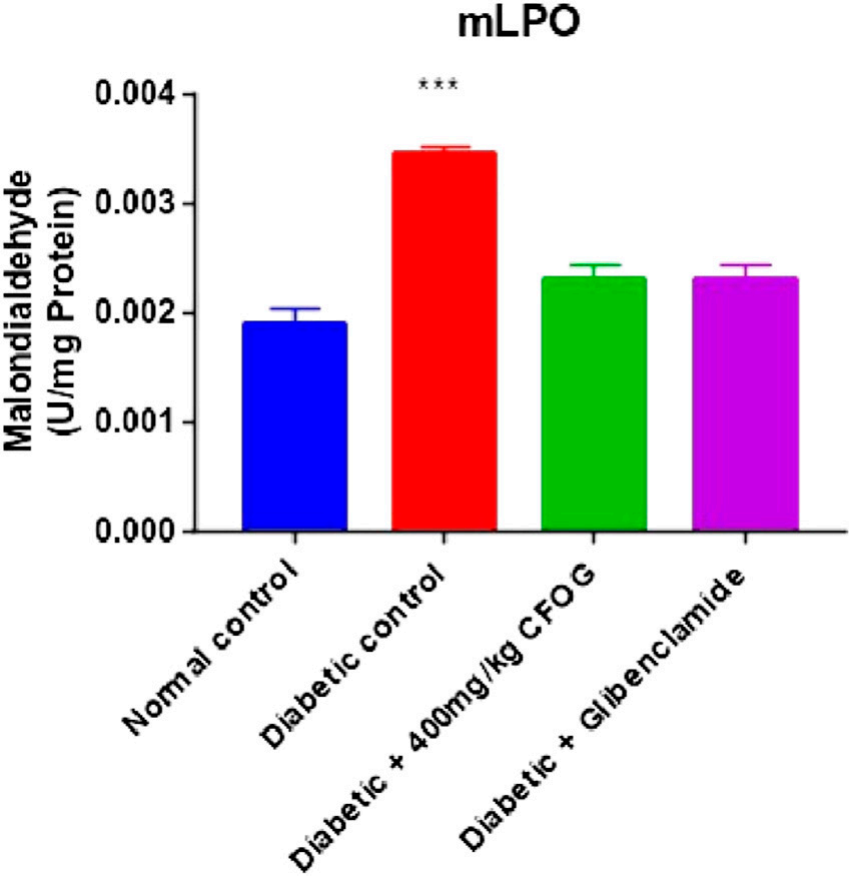
Effect of chloroform fraction of OG leaf on lipid peroxidation in type 2 diabetic rats.

One of the diabetogenic mechanisms of STZ is generation of free radicals which could lead to lipid peroxidation measured as malondialdehyde (MDA) in this study. The impact of CFOG on lipid peroxidation in STZ-induced diabetic rats was displayed in Figure 6. It was observed that only the diabetic control showed highly significant difference in MDA level relative to normal control. In contrast, MDA produced in other groups were similar to normal control group.

### 3.5 Investigation of the CFOG leaf effect on mATPase activity in diabetic rats

Table 3 represents the effect of CFOG on mitochondrial ATPase function in STZ-triggered diabetic rats. Diabetic control was observed to display highly significant difference in the enzyme activity compared to normal rats. However, 400 mg/kg CFOG and 5 mg/kg glibenclamide drastically reduced the enzyme activity relative to diabetic control.

**TABLE 3:** Assessment of the Effect of Chloroform Fraction of OG on mATPase Activity in STZ-induced Diabetic Rats.

Groups	micromole Pi/min/mg protein
Normal Control	0.018 ± 0.0^##^
Diabetic Control	3.4 ± 0.0**
Diabetic + 400 mg/kg CFOG	0.42 ± 0.0*^, #^
Diabetic + 5 mg/kg Glibenclamide	0.62 ± 0.0*^, #^
UCP	5.8 ± 0.0***^#^

UCP: uncoupler.

*Means comparison of the test group with normal control.

#Means comparison other groups with diabetic control group.

mATPase: mitochondrial ATPase.

CFOG: chloroform fraction of *ocimum gratissimum* leaf extract.

### 3.6 Examination of the influence of CFOG on DNA fragmentation in diabetic rats model

**FIGURE 7:**
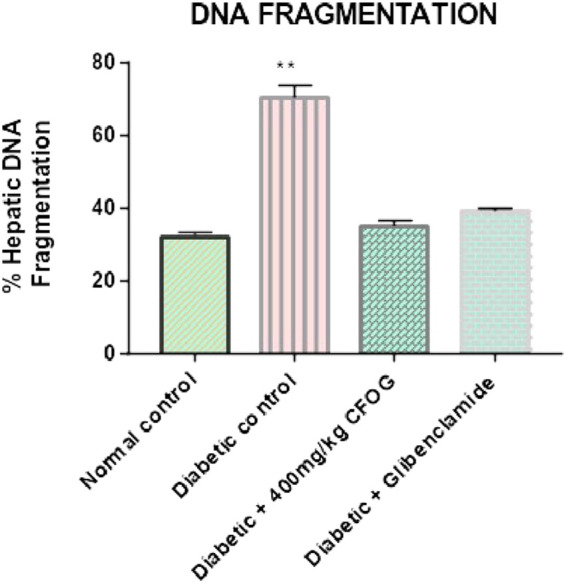
Effect of chloroform fraction of OG leaf on percentage DNA fragmentation in type 2 diabetic rats.

Figure 7 is an illustration of modulation of chromosomal DNA segmentation by CFOG in STZ-induced diabetic rats. Significantly high DNA fragmentation was observed in the diabetic control relative to normal control. Conversely, there was no significant difference in test groups, 400 mg/kg CFOG and 5 mg/kg glibenclamide in comparison with normal control.

### 3.7 Histological examination of pancreatic architecture of STZ-Induced diabetic rats treated with chloroform fraction of OG

**FIGURE 8:**
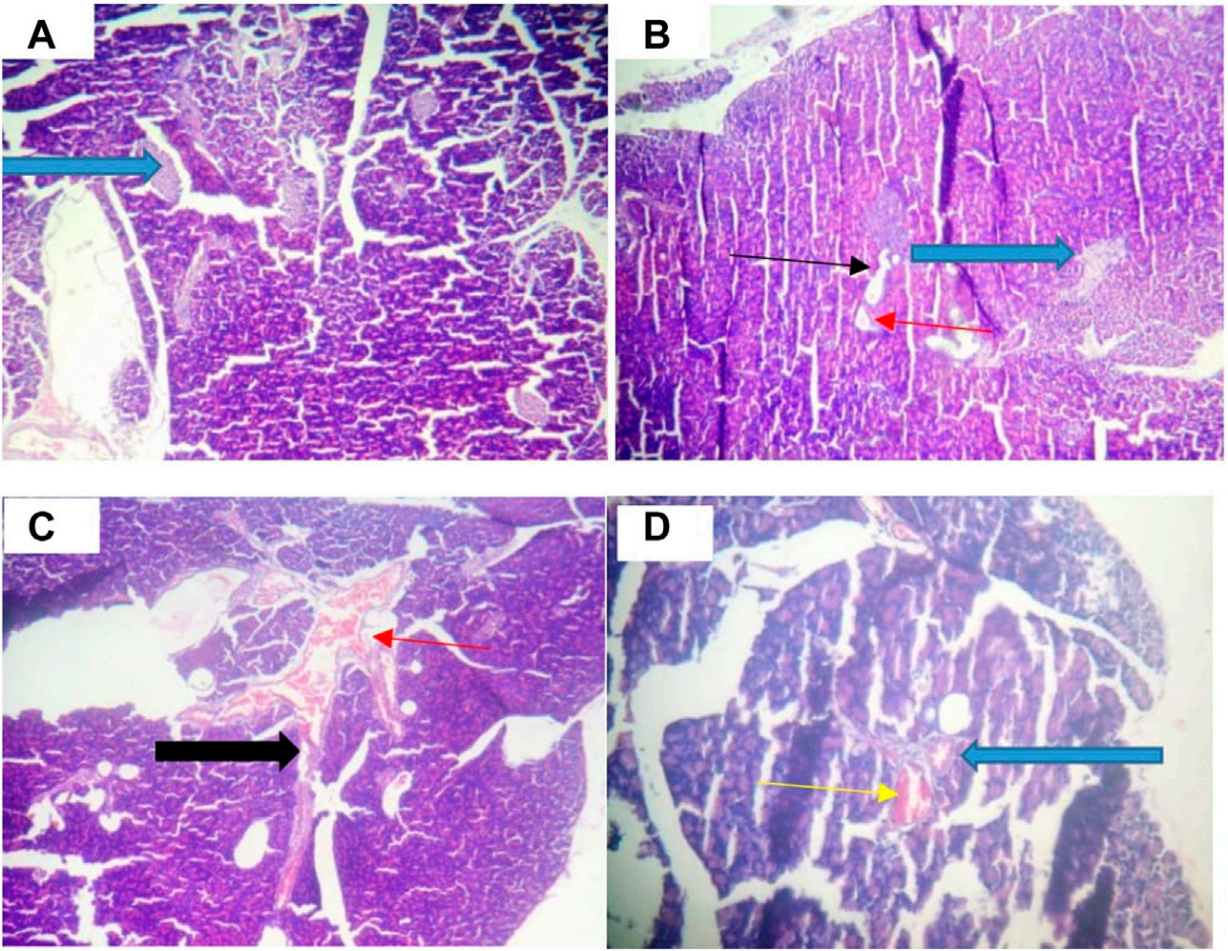
Pancreatic histology of STZ-induced diabetic rats treated with chloroform fraction of Ocimum gratissimum (X400).

Figure 8A shows that the pancreatic architecture was normal with exocrine acini (blue arrow) abundant and standard. The inter- and intra-lobular ducts were intact. Figure 8B depicts normal pancreas structure. Interlobular duct shows necrosis (black slender arrow) and haemorrhagic abrasion (red arrow). Figure 8C illustrates that exocrine acini filled with zymogen but the intralobular duct (black arrow) looked swelling and hyperemia (red) were noticed. In Figure 8D, undistorted exocrine acini (blue arrow), but intralobular distension and congestion (yellow arrow) were observed.

**FIGURE 9:**
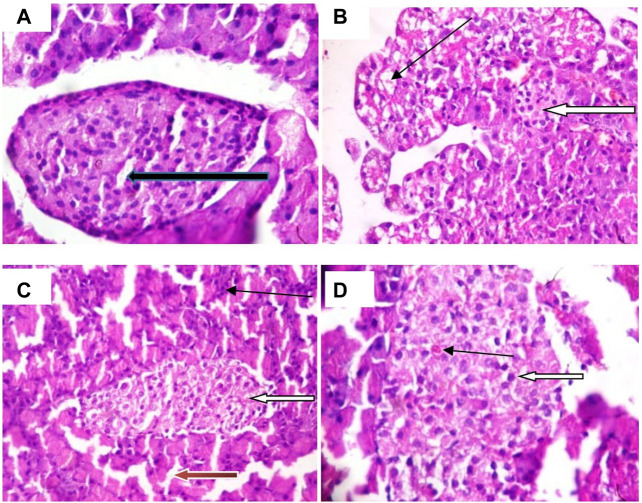
Photomicrograph of Islet cell of type 2 diabetic rats treated with chloroform fraction of Ocimum gratissimum leaf (X400).

Figure 9A illustrates that abundant number of islets was normally distributed within the parenchyma cells. Figure 9B shows that some islets of Langerhans appear atrophic (white arrow). The results in Figures 9C,D indicate normal islets of Langerhans (white arrow) consisting of round to oval collections of endocrine cells. Conversely, treatment with CFOG shows that it possesses phytometabolites that can repair damage islet cells.

### 3.8 Antioxidants status of STZ-induced diabetic rats treated with chloroform fraction of OG

**FIGURE 10:**
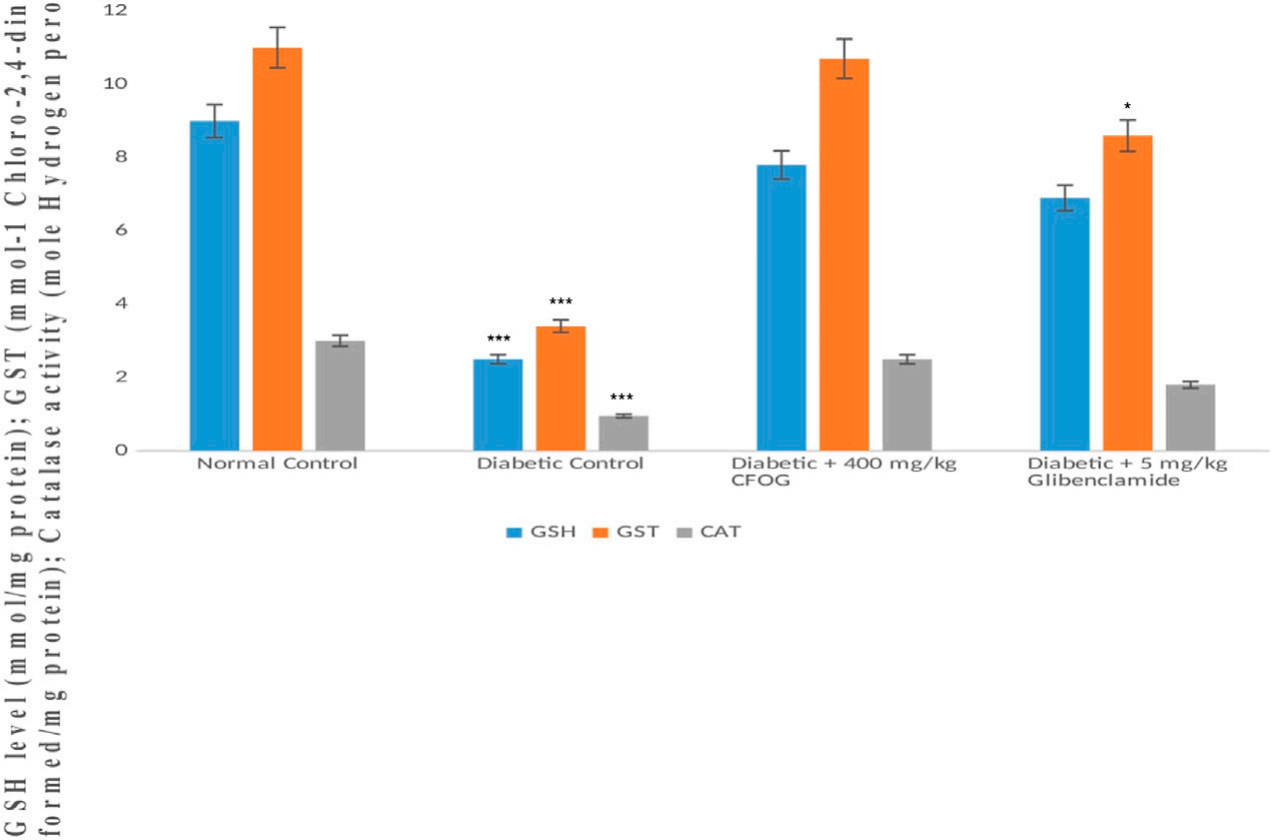
Assessment of the effect of CFOG on GSH level, GST and CAT activities in type 2 diabetic rats. *Means comparison of the test group with normal control.

GSH level, glutathione-S-transferase and catalase activities were displayed in Figure 10. The results showed that similar trends of significant decline were observed in the antioxidant status of diabetic control relative to normal control. Whereas, in the group treated with 400 mg/kg CFOG, there was no significant difference observed in the antioxidant enzymes activities and GSH level in comparison with the normal control.

### 3.9 Purified bioactive compound from chloroform fraction of OG

**FIGURE 11:**
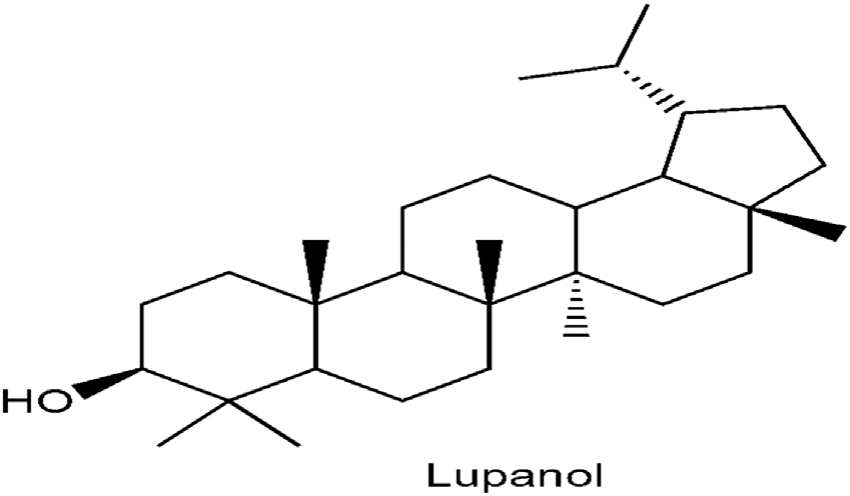
Characterisation of lupanol from *Ocimum gratissimum* (L.) leaf extract.

Figure 11 shows the structural elucidation of the bioactive principle which could be responsible for anti-diabetic agent. Lupanol was characterized from *O. gratissimum* leaf extract.

## 4 Discussion

Mitochondria are very vital organelles to the existence and survival of the cells as they are useful for energy production and other biochemical processes. Because of these salient roles, they are highly gated to prevent ‘intruders’ that could hamper their efficient functioning. Any condition that could lead to compromising the integrity of the two tight mitochondrial barriers would eventually collapse the organelles and the whole cell. The mitochondria breakdown could result from membrane potential dissipation and oxidative stress among others.

From the results above (Figure 4), the observed constancy of absorbances in the mitochondria suspension without triggering agent (calcium) and large amplitude pore opening in the suspension challenged with calcium in separate experiments depict that the membrane integrity has not been compromised. Subsequent reversal of the calcium-induced opening by spermine (a potent pore inhibitor), in another experiment over a period of 12 min at 30 s interval, in the presence of rotenone and succinate, further substantiates that the mitochondria were intact, not uncoupled and suitable for further use. This experiment is very important because the integrity of the mitochondrial membrane is crucial for ATP synthesis. In other words, mitochondria with lost membrane integrity cannot synthesize energy.

Diabetes mellitus is a derangement in carbohydrates, lipid and proteins metabolism. This pathophysiological condition is associated with inflammation, loss of integrity of beta cell mitochondria, elevated blood insulin and glucose, increased and overexpressed apoptosis. In the results obtained from the impact of CFOG on STZ-induced diabetic rats, normoweighted condition (Figure 1) was discovered in rats treated with the CFOG and this might have stemmed from its ability to attenuate tissue wastage related to DM due to the presence of phytochemicals that aid proper maintenance of macromolecules metabolism in the biological system. Glucose and insulin levels were also normalized by the administration of CFOG to diabetic rats (Table 2). This indicates that its bioactive metabolites could have played abrogating roles in normalizing the insulin and blood sugar status in the rats. Diets containing necessary antioxidants have been shown to demonstrate good anti-diabetic efficacy (Bacanli et al., 2016). Genistein, an isoflavone, significantly reduced glucose intolerance in diabetic rats (Lee, 2006). Tannin was reported to exhibit an anti-nutrient activity by inhibiting α-glucosidase, thus impeding or slowing down the absorption rate of glucose across the intestinal epithelial cell. Nutritive soy isoflavones drastically enhanced insulin biosynthesis thereby ameliorating excessive blood sugar and as well mitigated diabetic complication such as cataracts. Anti-hyperglycemic effect of low dose quercetin and quinic acid has been well documented (Arya, et al., 2014). *Beta vulgaris* was reported to lower blood glucose (Bolkent, et al., 2009). Similarly, complete abrogation of insulin resistance and considerable improvement in beta cell biomass were shown in Figures 3, 4 respectively. This depicts that CFOG could inhibit desensitization of insulin and probably its receptor too. Myriads of phytoactive molecules known to exhibit antioxidant potentials play crucial role in enhancing alertness and response of insulin in a condition of elevated glycemia (Bacanlia et al., 2019). Procyanidin from blueberry was also shown to reduce insulin resistance by mimicking this protein and also increase sensitivity through correction of the perturbed circulation of lipids and carbohydrate in the system (Yamashita et al., 2012).

Permeabilization of mitochondrial inner barrier to molecules of greater than 1.5 kDa, which leads to cell demise, is prominent in diseases associated with tissue wastage. STZ had 8.5folds pore induction and there was subsequent reversal of this, by 400 mg/kg CFOG (90%) as shown in Figure 5. This may suggest the ability of the fraction to maintain the viability of the rat liver mitochondria and as well block the triggering of pore formation associated with apoptosis. It is also coupled with obliteration of lipid peroxides (Figure 6) and prevention of the activation of ATPase activity (Table 3). Absence of mDNA fragmentation (Figure 7) in the diabetic rats treated with CFOG is an evidence that cell death did not occur. Similarly, histological examination of pancreas showed intact exocrine acini, normal and abundant (Fan et al., 2017; Baburina et al., 2019) islets in the diabetic rats administered CFOG as opposed to severely damaged pancreatic architecture in the diabetic control rat (Figures 8, 9). The presence of the phytometabolites in this solvent fraction could be liable for the anti-apoptotic properties observed in this experiment. Research substantiated that polyphenols boost mitochondria biogenesis and prevent mitochondrial insult (Park, et al., 2012). They also promote viability and survival of cells in conditions such as aging, neurodegenerative diseases and diabetes mellitus (Lionaki et al., 2015).

Moreover, our antioxidant results showed increase in the enzymes activities such as GST (phase II detoxifying enzyme), and CAT in the CFOG treated group as against the diabetic control rats (Figure 10). The similar elevation was also observed in non-enzymic endogenous antioxidant, GSH in the group administered 400 mg/kg CFOG (Figure 10). This may be owing to the availability of phytochemicals in the extract. Many researches have implicated oxidative stress in the pathogenesis of diabetes mellitus stemming from excess blood glucose and lipids (Dembinska-Kiee et al., 2008). It has been reported that in DM and its attendant complications such atherosclerosis and cardiovascular disorder, there is an obvious reduction in the plasma levels of vitamins C and E, zeaxathin, beta-carotene and lycopene, showing that overwhelming free radicals and oxidative stress are culprits in precipitating these diseases (Cheng et al., 2014; Girones-Vilaplana et al., 2014).

Vegetables and fruits which are usually rich in these phytonutrients are mostly recommended by the medical practitioners for the management of DM in order to ward off pro-oxidants generated in the course of the disease and to block its complications (Braun and Venter, 2008). Polyphenols are severally reported to profoundly prevent the onset of chronic ailments which are associated with oxidative imbalance in the cell. Due to the presence of phenolics, mulberry fruit was discovered to possess neuro-preserving and antidiabetic potency (Wang et al., 2013).

In this study, lupanol (Figure 11), a penta triterpene, was discovered to be present in *O. gratissimum* leaf using NMR technique. Terpenes have been implicated to possess anti-diabetic effect (Jovanovic et al., 2021). Research showed that 23, 28-dihydroxyl lupan-20 (29) ene 3β caffeat obtained from *Sorbus decora* which is structurally close to lupanol, was discovered to improve glucose uptake in C2Cl2 skeletal muscle cell line. *Momordica charantia* contains two terpenes such as 3β, 25-dihydroxyl-7β, 25- trimethoxy cucurbita-5, 23- (E)-diene and 3β, 25-dihydroxyl-7β, 25- trimethoxy cucurbita-5, 23- (E)-dien-19-al. These bioactive agents exhibit their functions as insulin sensitizers which could help reduce insensitivity of insulin to its receptor (Panigrahy et al., 2020).

Furthermore, corosolic acid isolated from *Lagerstroemia speciosa* acts as α-glucosidase inhibitor, while lupeol obtained from mango leaf significantly scavenge ROS and thus reduced oxidative stress in albino mice (Panigrahy et al., 2020).

## 5 Conclusion

It could be inferred from the above data that chloroform fraction of *O. gratissimum* (L) leaf extract prevents programmed cell death as evident in its ability to inhibit pore-opening, ATPase activity and lipid peroxidation in STZ-induced diabetic rats which could lead to tissue wastage experienced in diabetes mellitus. It could also be suggested that chloroform fraction probably contains bioactive principles which was able to scavenge free radicals generated in STZ-induced diabetic rats as observed in the antioxidant status of the extract-treated rats. The leaf fraction was also able to lower blood glucose, homeostasis model assessment of insulin resistance and enhance homeostasis model assessment of pancreatic beta cell. This shows the plant fraction has inherent metabolites which could act as insulin sensitizer and secretagogues. The presence of lupanol may be one of the bioactive compounds responsible for anti-diabetic activity of *O. gratissimum* leaf.

## Data Availability

The original contributions presented in the study are included in the article/supplementary material, further inquiries can be directed to the corresponding author.
